# Arsenic and high affinity phosphate uptake gene distribution in shallow submarine hydrothermal sediments

**DOI:** 10.1007/s10533-018-0500-8

**Published:** 2018-09-20

**Authors:** Ernest Chi Fru, Nolwenn Callac, Nicole R. Posth, Ariadne Argyraki, Yu-Chen Ling, Magnus Ivarsson, Curt Broman, Stephanos P. Kilias

**Affiliations:** 1 Department of Geological Sciences and Bolin Center for Climate Research, Stockholm University, 106 91 Stockholm, Sweden; 2 College of Physical Sciences and Engineering, School of Earth and Ocean Sciences, Geobiology Center, Cardiff University, Park Place, Cardiff, Wales CF10 3AT UK; 3 Department of Biology, Nordic Center for Earth Evolution (NordCEE), Campusvej 55, 5230 Odense M, Denmark; 4 Department of Geosciences & Natural Resource Management, Geology Section, University of Copenhagen, Øster Voldgade 10, 1350 Copenhagen K, Denmark; 5 Department of Geology and Geoenvironment, National and Kapodistrian University of Athens, Panepistimiopolis Zographou, 157 84 Athens, Greece; 6 Department of Biology, University of Southern Denmark, Campusvej 55, 5230 Odense M, Denmark

**Keywords:** Arsenic biogeochemistry, Arsenic speciation, Phosphate biogeochemistry, Hydrothermal activity

## Abstract

The toxicity of arsenic (As) towards life on Earth is apparent in the dense distribution of genes associated with As detoxification across the tree of life. The ability to defend against As is particularly vital for survival in As-rich shallow submarine hydrothermal ecosystems along the Hellenic Volcanic Arc (HVA), where life is exposed to hydrothermal fluids containing up to 3000 times more As than present in seawater. We propose that the removal of dissolved As and phosphorus (P) by sulfide and Fe(III)(oxyhydr)oxide minerals during sediment–seawater interaction, produces nutrient-deficient porewaters containing < 2.0 ppb P. The porewater arsenite-As(III) to arsenate-As(V) ratios, combined with sulfide concentration in the sediment and/or porewater, suggest a hydrothermally-induced seafloor redox gradient. This gradient overlaps with changing high affinity phosphate uptake gene abundance. High affinity phosphate uptake and As cycling genes are depleted in the sulfide-rich settings, relative to the more oxidizing habitats where mainly Fe(III)(oxyhydr)oxides are precipitated. In addition, a habitat-wide low As-respiring and As-oxidizing gene content relative to As resistance gene richness, suggests that As detoxification is prioritized over metabolic As cycling in the sediments. Collectively, the data point to redox control on Fe and S mineralization as a decisive factor in the regulation of high affinity phosphate uptake and As cycling gene content in shallow submarine hydrothermal ecosystems along the HVA.

**Electronic supplementary material:**

The online version of this article (10.1007/s10533-018-0500-8) contains supplementary material, which is available to authorized users.

## Introduction

Arsenic (As) is ubiquitous in the marine and terrestrial biosphere, where it exists primarily as inorganic arsenate-As(V), arsenite-As(III) and as a variety of arsenic sulfides (As-S) in oxic, anoxic and euxinic environments, respectively (Maher and Butler 1988; Cullen and Reimer 1989; Smedley and Kinniburgh 2002; O’Day et al. 2004; O’Day 2006; Henke 2009; Godelitsas et al. 2015). The ratio of As(III) to As(V) in oxygenated seawater is estimated to be ~ 10^−27^ and rapidly increases with deoxygenation (Maher and Butler 1988; Smedley and Kinniburgh 2002). This has resulted in the spread of As resistance mechanisms in the simplest to the most complex organisms inhabiting both oxic and anoxic habitats exposed to As(V) and As(III). Organic forms of As mostly found in highly productive waters, are believed to decrease and enhance toxicity at various instances (Wilkin et al. 2003; Hoffmann et al. 2018; Chen et al. 2017), but are also thought to be quantitatively negligible relative to As(III) and As(V) enrichment in the natural environment (Smedley and Kinniburgh 2002).

The pervasive spread of As resistance genes in microbial lineages at the base of the tree of life (e.g., Gihring et al. 2003; Jackson and Dugas 2003), suggests exposure to As in the deep geological past (Chen et al. 2017; Tian and Luo 2017). This ancient interplay between life and As resulted in the biological innovation of resistance mechanisms whereby As(V) is reduced with unique cytoplasmic As(V) reductases to As(III), followed by extrusion via specific As(III) cell membrane protein transporters (Rosen 2002; Jackson and Dugas 2003; Cai et al. 2009; Dziubinska-Maciaszczyk et al. 2011; Rosen et al. 2011; Slyemi and Bonnefoy 2012a, b; Zhu et al. 2017). Importantly, the presence and expression of these detoxification pathways in recently evolved lineages, including the more complex eukaryotes (Mukhopadhyay et al. 2002; Rosen 2002; Harrington et al. 2008; Fu et al. 2009; Qin et al. 2009; Dziubinska-Maciaszczyk et al. 2011), highlights the fact that As continues to exert considerable pressure on modern biological organization.

Notably, the physical and chemical similarities shared between As(V) and phosphate has resulted in As(V) interfering with biological phosphate metabolism (Thiel 1988; Takahashi et al. 1990; Dyhrman and Haley 2011; Guo et al. 2011; Rosen et al. 2011; Tawfik and Viola 2011; Elias et al. 2012; Xu and Nussinov 2012). Consequently, low affinity membrane phosphate transporters (Pit) that constitutively transport phosphate into the cell in phosphate-replete settings (Lin et al. 2016), are incapable of distinguishing As(V) from phosphate (Elias et al. 2012). Chemolithoautotrophs and many marine Cyanobacteria inhabiting As-rich geothermal environments switch on a high affinity phosphate uptake system (Pst) when dissolved As(V) to phosphate ratios rise above a certain threshold (Dyhrman and Haley 2011; Elias et al. 2012). The Pst system is also expressed in phosphate-starved conditions, regardless of whether As(V) is present or absent (Thiel 1988; Guo et al. 2011; Karl 2014; Lin et al. 2016).

Despite the challenge of As toxicity, a peculiar but polyphyletic group of microorganisms conserve energy from As, by respiring As(V) to As(III) and by oxidizing As(III) back to As(V) in a variety of marine and terrestrial habitats (e.g., Mukhopadhyay et al. 2002; Smedley and Kinniburgh 2002; Oremland and Stolz 2003; Rosen 2002; Gihring et al. 2003; Jackson and Dugas 2003; Oremland and Stolz 2003; Saltikov and Newman 2003; Malasarn et al. 2004; Stauder et al. 2005; Silver and Phung 2005; Quéméneur et al. 2008; Cai et al. 2009; Fu et al. 2009; Henke et al. 2009; Newman et al. 2009; Qin et al. 2009; Dyhrman and Haley 2011; Dziubinska-Maciaszczyk et al. 2011; Rosen et al. 2011; Sánchez-Riego et al. 2014; Zhu et al. 2014, 2017; Gilhooly et al. 2014; Jiang et al. 2014; Rascovan et al. 2016).

A number of studies have identified and successfully characterized microbial communities thriving in hydrothermal vent fields impacted by some of the most elevated As concentrations (e.g., Dando et al. 1995; Bayraktarov et al. 2013; Giovannelli et al. 2013; Price et al. 2013a, b; Ruiz-Chancho et al. 2013; Gilhooly et al. 2014; Godelitsas et al. 2015; Callac et al. 2017). Nonetheless, a mechanistic understanding of how life copes with high As conditions, the chemical and physical processes that regulate dissolved As content, and the potentially biolimiting phosphate concentrations caused by coprecipitation with hydrothermal Fe(III)(oxyhydr)oxides (e.g., Feely et al. 1996, 1998; Wheat et al. 1996; Schaller et al. 2000; Hawkes et al. 2014), remain to be clarified for these environments.

**Fig. 1:**
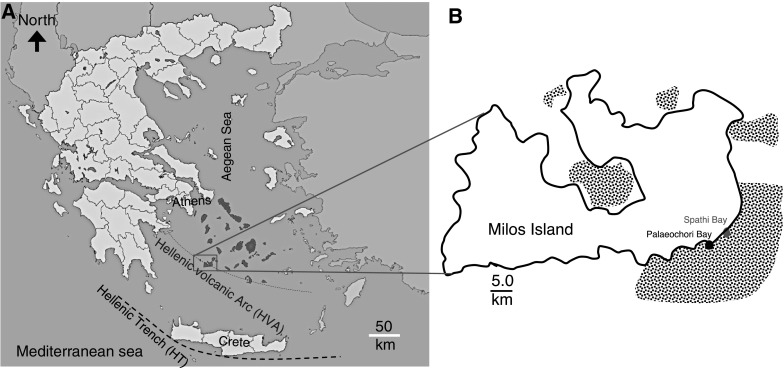
Location of field site in the Aegean Sea. **a** Location of Milos along the Hellenic Volcanic Arc (HVA). **b** Sampling site. Dots are areas where shallow submarine activity has been located on the seafloor (Dando et al. 1995)

Here, we demonstrate a link between two types of reactive minerals, Fe sulfide and Fe(III)(oxyhydr)oxides, and the scavenging of As and P across a transect of hydrothermally influenced sediments at Spathi Bay, on the coast of Milos Island, Greece (Fig. 1). In situ biogeochemical data are synthesized into a quantitative field-wide community composition model for the abundance of respiratory As(V) reductases (*arrA*), As(III) oxidases (*aoxB*), the As(III) membrane extrusion genes (*arsB*, *acr3*-*1* and *acr3*-*2*) and high affinity phosphate uptake (*pstB*) gene content, relative to S and Fe mineralogy.

## Study site

Spathi Bay is located at 36°40′N, 24°31′E in the south east of Milos Island, on the Hellenic Volcanic Arc (HVA), in the Greek Cyclades of the Aegean Sea (Fig. 1). Hydrothermal activity along the HVA is a result of the subduction of the African Plate underneath the Euroasian Plate (Kilias et al. 2013). The coast of Milos is recognized as one of the largest active, shallow submarine hydrothermal areas on Earth (Dando et al. 1995; Bayraktarov et al. 2013; Giovannelli et al. 2013; Price et al. 2013a, b; Ruiz-Chancho et al. 2013; Chi Fru et al. 2013; Gilhooly et al. 2014; Godelitsas et al. 2015), covering an estimated ~ 35 km^2^ of the shallow seafloor (Price et al. 2013b) (Fig. 1). Here, hydrothermal activity is accompanied by the emission of H_2_S-CO_2_-As-rich fluids, along the entire HVA (Bayraktarov et al. 2013; Giovannelli et al. 2013; Price et al. 2013a, b; Ruiz-Chancho et al. 2013; Chi Fru et al. 2013; Gilhooly et al. 2014; Godelitsas et al. 2015; Callac et al. 2017), with ferruginous sediments dating back ~ 2.0 million years characterized by a high As anomaly (Chi Fru et al. 2013, 2015b, 2018).

**Fig. 2:**
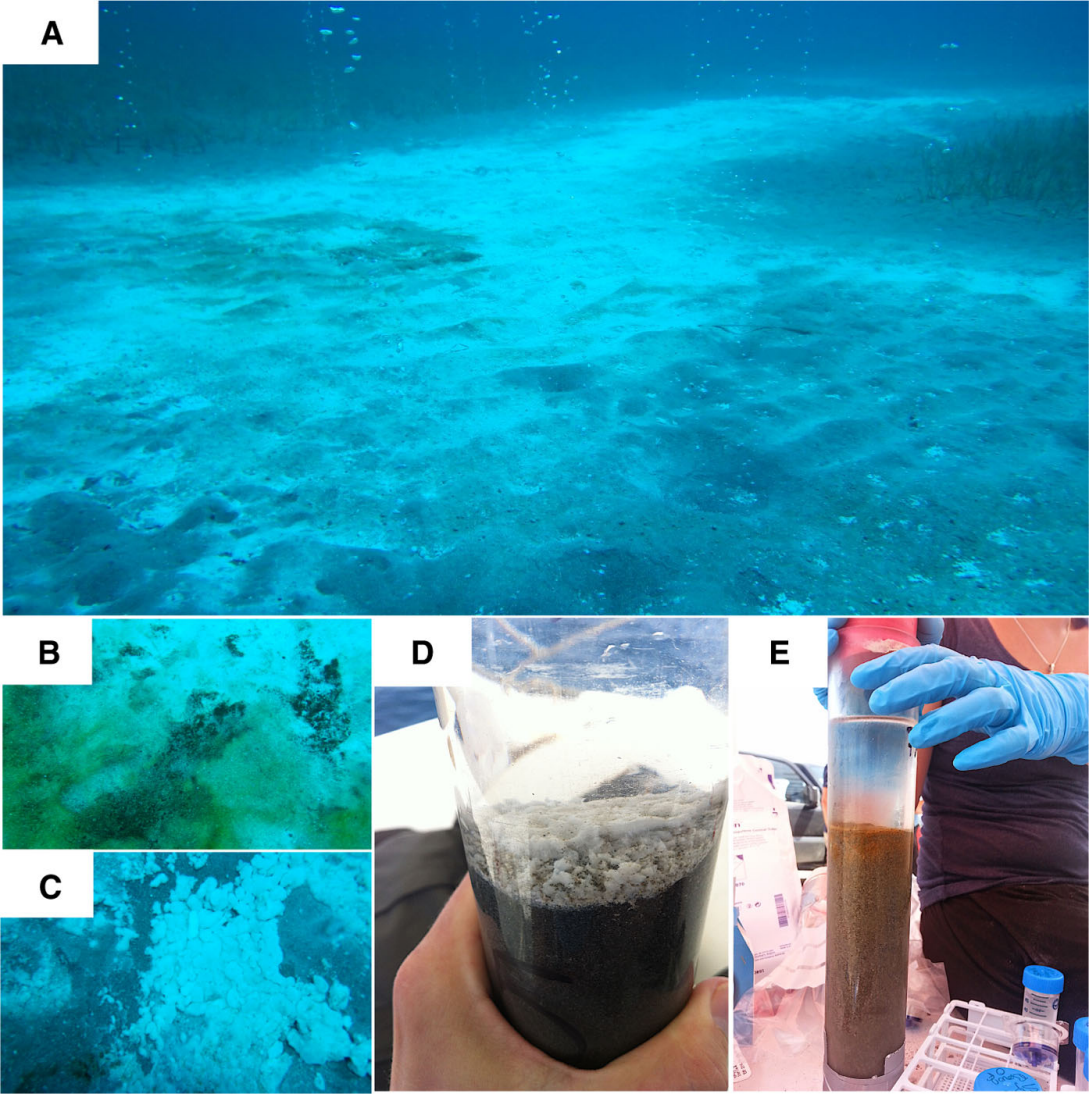
Sample collection. **a** Seafloor diffused hydrothermal activity characterized by gas bubbles and the seafloor white and brown mats. **b** Close up view showing the sharp boundary between the white and brown seafloor deposit. **c** Close up view of white deposit on the seafloor. **d** Push core sample for the white-capped sediment. **e** Push core sample for the brown-capped sediment

Hydrothermal fluid emission on the shore of Milos Island has temperatures, pH, salinity and H_2_S concentration reaching 40–116  °C, 5.0, 50% and 3.1 mmol l^−1^, respectively (Bayraktarov et al. 2013; Giovannelli et al. 2013; Price et al. 2013a, b; Ruiz-Chancho et al. 2013; Gilhooly et al. 2014; Godelitsas et al. 2015). Fluids emitted through unconsolidated sandy sediments produce up to 2.9 × 10^3^ ppb As at Palaeochori Bay and 5.9 × 10^3^ ppb As at Spathi Bay (Giovannelli et al. 2013; Price et al. 2013a, b; Ruiz-Chancho et al. 2013). Indeed, the Spathi Bay hydrothermal fluids are up to ~ 3000 times richer in As than local seawater, making them some of the richest As fluids known for any hydrothermal emissions on Earth (Breuer and Pichler 2013). The anoxic hydrothermal solutions mixing with oxygenated seawater contain mainly ~ 49%-As(III) and 9%-As(V), the remainder identified as various dissolved thioarsenic sulfide species (Giovannelli et al. 2013; Price et al. 2013a, b; Ruiz-Chancho et al. 2013). These fluids precipitate concentric, colorful, sand-grain coatings around the vent centers, and are broadly characterized by yellow-orange As sulfide patches, white sulfur-silica biomats, and outward-bound brown-capped sediments comprised mostly of Mn oxides and Fe(III)(oxyhydr)oxides sand coatings (Dando et al. 1995; Kilias et al. 2013; Giovannelli et al. 2013; Price et al. 2013a, b; Ruiz-Chancho et al. 2013; Callac et al. 2017; Fig. 2). Sand-capped sediments, which lack gas bubbles, seafloor hydrothermal seeps and the colorful sand coatings and biomats, rim the active venting centers (Callac et al. 2017).

## Methods

### Sampling

Seawater samples were collected at 1 m intervals down to the seafloor depth of 12.5 m, using a Niskin Bottle that was thoroughly rinsed with water from the sampled-depth, 500 ml of which was filtered through 0.22 µm nylon membrane filters (Maine Manufacturing). In addition, 25 ml unfiltered seawater was mixed with 96% ethanol in 50 ml Falcon^®^ tubes. All samples were immediately frozen and transported on dry ice for molecular analyses. Replicate push core samples were collected and processed as previously described (Callac et al. 2017). Briefly, in a field anaerobic glove bag (Captair^®^ Pyramid glove Bag, Cole-Parmer), under a N_2_ atmosphere, sediment cores were sectioned with sterile disposable plastic spatulas into 2 cm slices and stored in 50 ml Falcon^®^ tubes. The tubes were immediately frozen on dry ice, shipped to the laboratory and stored at − 20 °C, together with the water samples. Samples for chemical analysis were put in sterile anoxic bags, flushed with N_2_ and preserved on dry ice during transportation.

### Sediment porewater and seawater chemistry

Seawater oxygen content, electrical conductivity, pH and temperature were measured during sampling with microprobes attached to the Niskin Bottle sampler. Sediment porewater for total trace element content was extracted under a nitrogen atmosphere from sectioned cores by centrifugation for 10 min at 6439.2 g (Callac et al. 2017). The recovered water was filtered through 0.2 µm polyethersulfone (PES) membrane filters (Sarstedt^®^) prior to analysis. Retrieved porewater was low for several samples. These samples, together with the overlying seawater samples, were acidified to a final HNO_3_ concentration of 0.28 mol l^−1^ with ultra-pure grade HNO_3_. The acidified solutions were further diluted 1000-fold in 0.28 mol l^−1^ HNO_3_ acidified milli-Q H_2_O and analysed by inductively coupled plasma-optical emission spectrophotometry (ICP-OES) for trace element composition. For porewater arsenite, arsenate and phosphate measurements, 10 g of wet sediment was diluted in 40 ml MQ-H_2_O in a COY anaerobic chamber. After equilibration, 1.0 ml of the settled solution was passed through a 0.2 µm filter and analysed by the molybdate blue complexation reaction (Johnson 1971; Johnson and Pilson 1972; Dhar et al. 2004). Absorbances were obtained at 865 nm wavelength for the color complex formed on a Cary 50 Probe UV–vis spectrophometer. Porewater concentrations were deduced from linear plots of known concentrations of arsenate and phosphate standards plotted against their absorbances with R^2^ = ~ 0.999 (Johnson 1971; Johnson and Pilson 1972; Dhar et al. 2004). Values were reported as averages of duplicate measurements after accounting for the dilution factor.

### Sediment mineralogy

Powder X-ray diffraction (PXRD), using a PANalytical Xpert-pro diffractometer, at room temperature, was used to analyse the mineralogical composition of sediments dried at 60 °C overnight and powdered in an agate mortar. The instrument was run at 45 kV and 40 mA at 1.5406 Å wavelength using Cu-Kα radiation and Ni-filter. Samples were run between 5 and 80° in step sizes of 0.017° and scan step time of 50.1650 s in continuous scanning mode while rotating samples. Raman spectroscopy was performed using a confocal laser Raman spectrometer (LabRAM HR 800; Horiba), equipped with a multichannel air-cooled charge-coupled device detector as previously described (Chi Fru et al. 2013, 2016c). Spectral resolution was ~ 0.3 cm^−1^/pixel. Accuracy was determined by a repeated silicon wafer calibration standard at a characteristic Raman line of 520.7 cm^−1^.

### Sediment reactive iron and reactive sulfide content

Sequential iron extraction was performed according to the method of Poulton and Canfield (2005) to separate six distinct Fe phases: (1) Fe carbonates, (2) Fe(III)oxyhydroxides, (3) magnetite, (4) hematite, some goethite and akaganéite, (5) poorly reactive sheet silicate Fe, and (6) total sediment Fe. These analyses were performed by sequential treatment of 50 mg of oven-dried (60 °C) and powdered samples with 10 M sodium acetate, sodium dithionite, ammonium oxalate, a second sodium dithionite extraction, followed by boiling in 12 M HCl, and total dissolution with heat, nitric and hydrofluoric acid treatment, respectively (Poulton and Canfield 2005). Total Fe in these six fractions was measured by colorimetric UV–vis spectrophometry using the ferrozine reaction (Stookey 1970; Viollier et al. 2000). Total sediment sulfide content was estimated following extraction by the cold chromium distillation method (Kallmeyer et al. 2004) to account for both pyrite and acid volatile sulfide, according to Cline and co-workers (Cline 1969).

### Molecular analysis

DNA extraction was performed in duplicates on ~ 0.25 g of sediment, using the Mo Bio PowerSoil DNA kit (Carlsbad, CA) according to the manufacturer’s instructions. Seawater DNA stored on filters was extracted using the MoBio PowerWater^®^ DNA Isolation Kit (Carlsbad, CA) following the manufacturer’s instructions. DNA from the seawater preserved in 96% ethanol solution was extracted, after centrifugation at 10,732 g for 30 min, using the DNeasy^®^ Blood and Tissue kit (Qiagen) as specified by the manufacturer.

Using a universal primer set targeting the *aoxB* and *Geobacteraceae*-specific primers for the *arrA*, *acr3*-*1, acr3*-*2* and *pst* genes and 16S rDNA (Table S1), qPCR was performed in 96-well plates in a CFX96 Touch™ Real-Time PCR Detection System (C1000 Touch™ Thermal, Cycler, Bio-Rad). The *arrA* and *aoxB* genes carry out respiratory As(V) reduction and As(III) oxidation, respectively, in prokaryotes (Oremland and Stolz 2003; Páez-Espino et al. 2009; Song et al. 2009; Kumari and Jagadevan 2016). The *acr3*-*1* and *acr3*-*2* genes, common in both prokaryotes and eukaryotes and the prokaryotic-specific *arsB* gene, code for efflux proteins that pump As(III) out of cells during As detoxification (e.g., Oremland and Stolz 2003). The *pst* gene codes for high affinity phosphate uptake in microbial organisms and has been shown to be especially important for survival in As-rich phosphate-poor geothermal environments (e.g., Elias et al. 2012).

Individual gene quantification by qPCR was in final volumes of 25 µl, using the SsoAdvancedTM Universal SYBR^®^ Green Supermix (Bio-Rad), following the manufacturer’s recommendations. Samples contained 5 µl of DNA (1 ng/µl), specific primer set at appropriate concentrations and annealing temperatures (Table S1), in 35 cycle reactions. Standard curves were calibrated using ten-fold dilutions from pure cultures (Table S1). qPCR gene quantification was performed in triplicates in samples, standards, together with negative controls to check for laboratory contamination. The total gene copy numbers per gram of sediment or per litre of seawater was calculated from the triplicate sample averages as previously described (Callac et al. 2015, 2017). *Geobacteraceae*16S rRNA gene abundance was estimated according to the average of 4.1 for bacteria 16S rRNA genes per cell (Lee et al. 2009; Callac et al. 2015, 2017). We assumed one copy per genome for the As and phosphate genes. Standard curves of ten orders of magnitude, determined from serially diluted DNA from pure cultures and quantified by NanoDrop spectrophotometry, were used to calculate gene abundances. The accepted efficiency of the qPCR data was determined to be ≥ 95% from the slope of the standard curves.

### Statistical analysis

Multivariate statistical analysis was performed using the Minitab 17 statistical software with the aim to explore the association of genomic with sediment geochemical data. Principal component factor analysis with a varimax rotation was applied to the total dataset in order to create factors, each representing a cluster of interrelated variables. Factor analysis and cluster analysis using the average neighbor linkage measure were applied to the data after normal score transformation of the raw values. This ensured the normal distribution for variables and reduced the influence of high values on the output results. The selection of the optimum principal components was based on the scree plot, which is the graphical visualization of the relationship between the eigenvalues and the number of components. In this case, the cut-off was chosen at the point where the function displayed by the scree plot showed an elbow, allowing for the division of the major components from the trivial components. The distance measure used in cluster analysis was the Pearson correlation coefficient at 95% confidence level.

## Results and discussion

### Water column and sediment chemistry

Temperature, electrical conductivity (EC), oxygen concentration and pH of seawater along the 12.5 m depth profile to the seafloor over the two-day sampling period, averaged 24 °C (SD ± 0.4, n = 35), 59.01 mS cm^−1^ (SD ± 0.07, n = 11), 9.4 mg l^−1^ (SD ± 0.16, n = 23) and 8.3 (SD ± 0.012, n = 11), respectively. The alkali and alkaline Earth metals K, Mg, Ca and Na, together with redox sensitive S, were the most abundant elements in the sediment porewater (Table S2). P and Pb were low in all sediment types, depth and seawater, with P being predominantly below the ICP-OES detection limit of 2.0 ppb in the sediment porewater and overlying seawater. The abundance of K in the Milos hydrothermal sediments has previously been attributed to silicate mineralization (Price et al. 2013a).

**Fig. 3:**
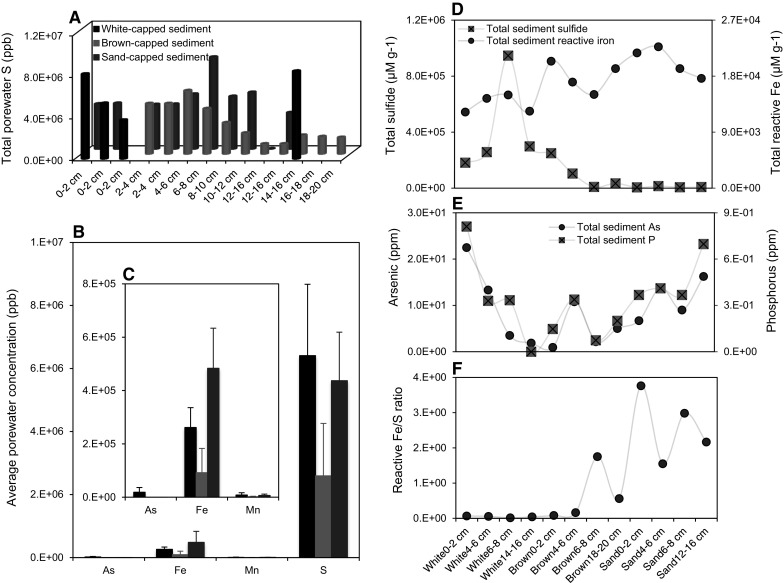
Total sediment sulfide obtained by the Cr distillation method and average porewater ICP-OES concentrations of S, Mn, Fe and As in sediment core down to 20 cm. **a** Total porewater sulfur versus depth. **b** Average porewater S, Mn, Fe and As. **c** Magnification of average porewater Mn, Fe and As content from (**b**). Bars are standard deviation from the mean. **d** Total sediment sulfide and Fe versus depth and habitat. **e** Total sediment As and P versus depth and habitat. **f** Total reactive Fe (Fe(III)(oxyhydr)oxides) to total reactive sediment sulfide content plotted against habitat and sediment depth

The highest porewater total As content (42.7 ppb) was recorded in the white-capped sediment at 14–16 cm depth, while concentrations in the brown/sand-capped sediments were below detection at some depths, reaching a maximum of 0.6 ppb in the brown-capped sediments. Despite elevated sediment As concentration, seawater content above the sampled sediments was below the ICP-OES detection limit, with the exception of one positive outlier (Table S2). The trace metals Cd, Cr and the biologically important trace nutrients, Co, Mo, Cu, Fe, V and Ni were depleted in the seawater column. The depletion of trace metals and nutrients was similar in the sediments. Cd was only detected in the white-capped sediments, while detectable Mo was mainly recorded in the brown- and sand-capped sediments. Below 10 cm, Co was not detected in the brown-and sand-capped sediments. S was present in all samples and depths, including seawater, but decreased dramatically with depth in the brown-capped sediment, while the deepest section in the white-capped sediment that could be analysed, sustained high levels (Fig. 3a). However, porewater chemistry for the intermediate depths in the white-capped sediments could not be measured by ICP-OES, because too little water could be retrieved for analysis.

Comparative analysis suggests average porewater S content is several orders of magnitude above As, Fe and Mn concentrations in all sediment types, being most elevated in the white-capped sediment (Fig. 3b–c). An above and below average positive linear correlation emerged between porewater As and S in the sulfide-rich and sulfide-depleted white-capped and brown/sand-capped sediments, with R^2^ values of 0.62538 and 0.45066, respectively. These relationships indicate a ~ 63 and 45% variation in the sedimentary As content, related to S in the white-capped and brown/sand-capped sediments, respectively. A previously unknown robust positive correlation was observed between porewater As and Mn concentrations in all habitats (R^2^ = 0.86248, 0.88342 and 0.96749) for the white-, brown- and sand-capped sediments, corresponding to ~ 86, 88 and 97% co-variation between the variables, respectively. However, a linear correlation for porewater As and Fe became apparent only in the sand-capped sediments, with R^2^ values increasing in the order of 0.03404, 0.46744 and 0.93176 for the white-, brown- and sand-capped sediments, respectively. These values, which correspond to 3, 47 and 93% related variation between As and Fe along the sampled transect, suggest porewater in the three sediment types are chemically distinct, in agreement with previous findings (Callac et al. 2017).

### Sediment sulfide and Fe(III)(oxyhydr)oxides mineralization

**Fig. 4:**
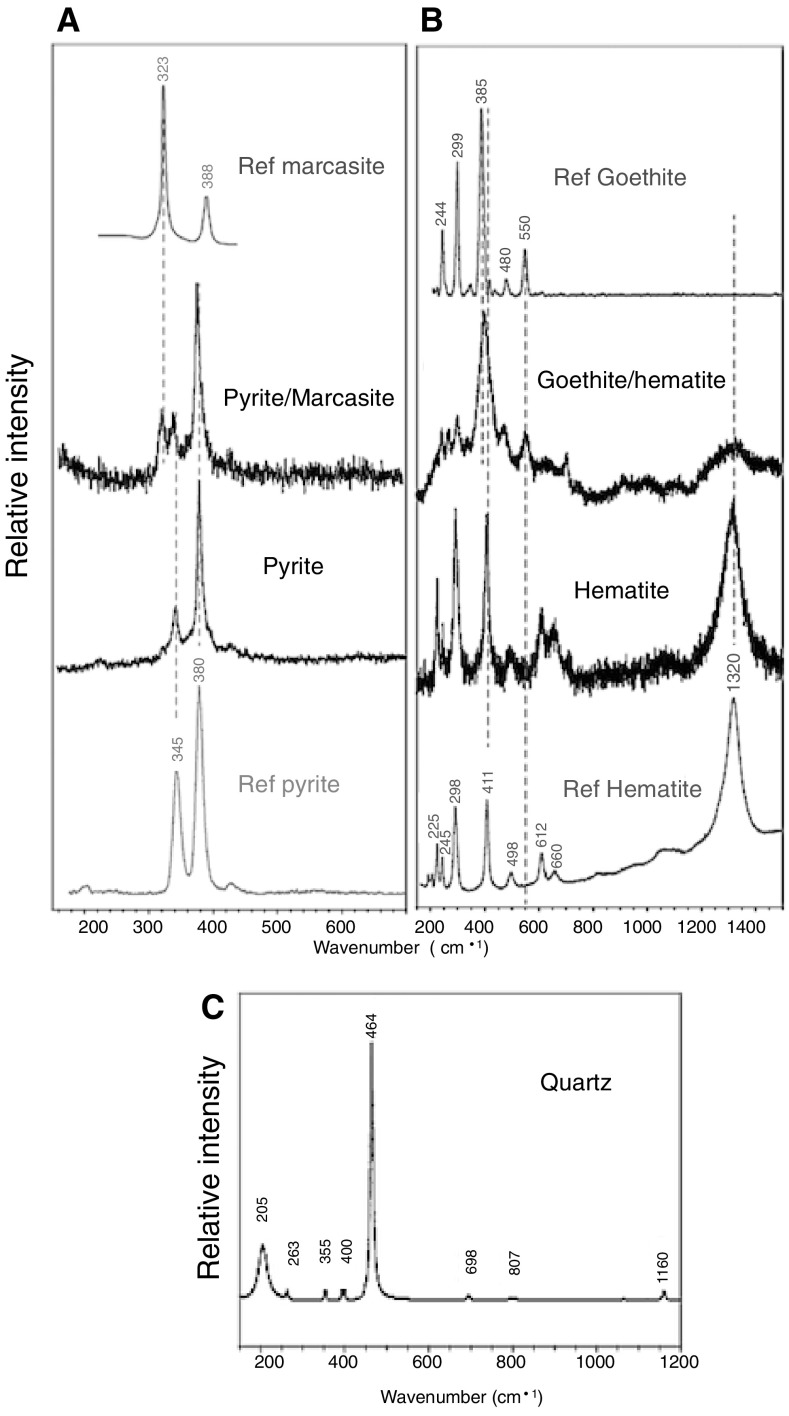
Raman mineralogical analysis. **a** Raman spectra for marcasite intergrowth in pyrite in the white-capped sediment. **b** Raman spectra for Fe oxides phases. **c** Raman spectrum for quartz

Maximum total sediment sulfide concentration across the study transect reaches 9.5 × 10^5^ μM g^−1^ of sediment at 6-8 cm in the white-capped deposit (Fig. 3d), consistent with a black sulfide color (Fig. 2d), odour and the detection of pyrite and marcasite by Raman spectroscopy (Fig. 4a). Sulfide declines with depth in the brown-capped sediment, similar to total porewater S concentration (Fig. 3a, d). A decrease in average sulfide content by 4.2 and 48.1 times in the brown- and sand-capped sediments relative to the white-capped sediment, respectively, suggests a declining sulfide front away from the hydrothermal center (Fig. 3d). Moreover, while pyrite and marcasite are below detection in the brown and sand-capped sediments, Fe(III)(oxyhydr)oxides concentration generally increases from the white-capped deposit to the sand-capped setting (Fig. 3d).

Total sediment As and P along this sulfide gradient, covary with a linear correlation fit of R^2^ = 0.82 (Fig. 3e). A similar observation in the Pacific Ocean water column (Santosa et al. 1997), hints that the global sediment As and P cycles are not mutually exclusive. Calculated reactive S and Fe ratios (Fig. 3f) indicate that a robust inverse relationship between sulfide and Fe(III)(oxyhydr)oxides production plays a key role in As and P mobility across the studied transect. As-sulfides, and up to 1.3 wt% Fe(III)(oxyhydr)oxides have been reported in these sediments (Price et al. 2013b; Ruiz-Chancho et al. 2013). Chemical extraction of Fe(III)(oxyhydr)oxides phases (Table 1), and hematite and potential goethite phases detected by Raman spectroscopy (Fig. 4b), suggest that Fe(III)(oxyhydr)oxides are a potent P sink, but a relatively weaker As sink in the sediments dominated by quartz (Fig. 4c). Although they vary in amplitude, the difference in the ratio of P to As associated with Fe(III)(oxyhydr)oxide distribution across the sediment types is nearly constant (Table 1), similar to their total sediment content (Fig. 3e).

**Table 1:** Fe, P and As content in ppm, associated with Fe phases, sediment type and depth

Sediment type	Depth (cm)	Fe carbonates			Oxyhydroxides			Magnetite			Hematite			Sheet silicates			Total extraction		
		Fe	P	As	Fe	P	As	Fe	P	As	Fe	P	As	Fe	P	As	Fe	P	As
White	0–2	210.18	0.56	0.82	227.69	0.67	1.88	30.65	0.41	nd	214.56	0.07	nd	599.88	0.45	nd	1695.11	0.81	22.49
White	2–4	223.31	1.04	nd	267.10	0.30	1.95	56.92	0.48	1.00	258.34	0.26	nd	722.48	0.30	0.78	1827.32	0.33	13.33
White	4–6	288.99	0.60	1.23	258.34	0.56	1.65	43.79	0.37	0.94	245.21	0.00	0.83	547.33	0.45	0.25	1687.80	0.33	3.51
White	6–8	185.25	0.61	0.45	239.99	0.32	0.83	46.31	0.47	0.64	218.93	0.21	0.12	509.44	0.32	0.34	1455.19	0	1.87
Brown	0–2	169.79	0.27	0.38	647.87	0.00	0.17	31.28	0.30	0.12	290.42	0.11	nd	688.08	0.65	1.50	1864.19	0.15	0.94
Brown	2–4	62.55	0.27	1.23	585.31	0.23	nd	31.28	0.15	1.17	272.55	0.15	1.68	545.10	0.42	0.69	1911.26	0.34	10.79
Brown	4–6	52.54	0.15	2.81	516.68	0.63	nd	21.89	0.22	1.30	249.58	0.04	1.41	604.26	0.52	nd	1845.49	0.07	2.14
Brown	6–8	47.22	0.77	2.07	746.95	0.73	1.34	21.46	0.51	0.89	257.57	0.11	0.06	639.63	1.06	2.18	nd	nd	nd
Sand	0–2	52.97	0.30	2.13	847.49	0.30	0.28	39.73	0.26	1.22	273.67	0.08	0.66	578.23	0.90	2.03	2055.21	0.37	6.70
Sand	2–4	71.49	0.08	1.52	884.67	0.27	nd	44.68	0.30	0.63	268.08	0.15	nd	634.46	0.57	0.84	2061.85	0.41	13.75
Sand	4–6	56.92	0.11	2.07	731.24	0.11	1.73	35.03	0.22	0.13	249.58	0.04	nd	608.64	0.71	1.79	1990.31	0.37	9.00
Sand	6–8	43.79	0.26	0.20	678.69	0.48	nd	17.51	0.22	nd	245.21	0.04	nd	477.28	0.22	0.29	1861.15	0.70	16.25

*nd* not detected

Of all the Fe(III)(oxyhydr)oxide phases, magnetite-like Fe content is low in all habitats (Table 1; Fig. S1). Potential Fe carbonates are ~ 5 times higher in the white-capped sediments than in the brown-/sand-capped sediments (Table 1), where they decrease with depth. To the contrary, Fe associated with Fe(III)oxyhydroxides are over 2–4 times greater in the brown-/sand-capped sediment than they are in the white-capped sediment, with the sand-capped samples having the most elevated concentrations (Table 1). Hematite, magnetite and sheet silicate Fe (phyllosilicate clay minerals) are evenly distributed throughout the various habitats and depths (Fig. S1).

Total sediment As content peaked close to the seafloor in the white-capped sediments, before decreasing with depth. For example, up to 12 times lower As concentrations are recorded at 6 cm than in the 0–2 cm depths (Table 1). No distinct trends were obvious in the brown-/sand-capped sediments. At no point did the estimated Phosphorus_sediment total_ to Fe(III)(oxyhydr)oxide_sediment total_ + Phosphorus_sheet silicate bound total_ ratios exceed 1, suggesting that most P in the sediments is adsorbed to Fe(III)(oxyhydr)oxides and clayey silicate minerals. To the contrary, Arsenic_sediment total_ to Fe(III)(oxyhydr)oxide_sediment total_ + Arsenic_sheet silicate bound total_ ratios were consistently > 1, indicating the presence of As sinks in the sediments other than Fe(III)(oxyhydr)oxides and sheet silicates. Our data, together with past mineralogical evidence, suggests these As sinks are most likely As-sulfides and Mn oxides. Both As sulfides and Mn oxides are known to prevail in this system and are discussed further below. XRD analysis revealed the ubiquity of the prominent marine Mn oxide mineral, birnessite, at all depths (Callac et al. 2017).

Indeed, Mn, Fe and S chemistry are the main chemical components that strongly influence environmental As mobility (Takamatsu et al. 1985; Cullen and Reimer 1989; Dando et al. 1995; Santosa et al. 1997; Ouvrard et al. 2002; Smedley and Kinniburgh 2002; Dixit and Hering 2003; O’Day et al. 2004; O’Day 2006; Maity et al. 2005; Lafferty et al. 2011; Breuer and Pichler 2013; Chi Fru et al. 2013; Dekov et al. 2013; Giovannelli et al. 2013; Gilhooly et al. 2014; Kilias et al. 2013; Price et al. 2013a, b; Ruiz-Chancho et al. 2013; Villalobos et al. 2014; Godelitsas et al. 2015). Mn oxides specifically oxidize As(III) and then adsorb and precipitate the resulting As(V) (Takamatsu et al. 1985; Ouvrard et al. 2002; Maity et al. 2005; Lafferty et al. 2011; Villalobos et al. 2014). Our data suggest a strong correlation between porewater Mn content and As, with the Mn oxide birnessite found at all sampled depths and habitats, recognized to be a strong As sink (Takamatsu et al. 1985). However, the consistent relationship that emerged between porewater Mn and As within and between habitats, implies that while Mn may be generally important for dissolved porewater As behaviour, it likely cannot account for As variability between sites.

Fe(III)(oxyhydr)oxides on their part are a well described As sink, resulting in their widespread application in the remediation of As-impacted waters (Cullen and Reimer 1989; Smedley and Kinniburgh 2002; Dixit and Hering 2003; Henke et al. 2009). There is therefore a general consensus that where Fe(III)(oxyhydr)oxides form, they coprecipitate As in its +3 and +5 oxidation states (Cullen and Reimer 1989; Smedley and Kinniburgh 2002; Henke et al. 2009; Kilias et al. 2013; Chi Fru et al. 2015a; Hemmingsson et al. 2018). The increase in Fe(III)(oxyhydr)oxides precipitation from the white-capped hydrothermal center to the bordering sand-capped sediment, counterbalanced by a drop in sulfide production (Fig. 3d, f), is therefore expected to be important for dissolved As mobility. The role of As sulfide precipitation in the global regulation of As mobility is less discussed, although reactive S/Fe ratios have been implicated as a possible indicator (O’Day et al. 2004; O’Day 2006). Environmental As and S chemistry are tightly coupled, such that sulfide production always results in the formation of As sulfides (Cullen and Reimer 1989; Smedley and Kinniburgh 2002; Stauder et al. 2005; Henke et al. 2009; Kilias et al. 2013; Chi Fru et al. 2015a; O’Day et al. 2004; O’Day 2006; Wilkin et al. 2003). Moreover, when sulfide production increases, it is commonly accepted that the accompanying precipitation of Fe sulfides hinder or outcompete the deposition of Fe(III)(oxyhydr)oxides (Canfield 1998; Poulton and Canfield 2011). This is consistent with the inverse relationship that emerged between total sulfide and Fe(III)(oxyhydr)oxides concentrations. In situ chemical data had long since shown that sulfide minerals quickly precipitate around hydrothermal vents, while Fe(III)(oxyhydr)oxides dispersal increases further away (Poulton and Canfield 2011; Chi Fru et al. 2015a). This is also coherent with our data.

In anoxic settings with pH < 6, As(V) is often preferentially coprecipitated with Fe(III)(oxyhydr)oxides at the expense of As(III) which preferably adsorbs onto Fe(III)(oxyhydr)oxides at pH > 7 (Dixit and Hering 2003). However, As(V) is not stable in sulfidic-anoxic environments where As(III) predominates (Cullen and Reimer 1989; Smedley and Kinniburgh 2002; Henke et al. 2009). This is the case for the sulfidic-anoxic hydrothermal fluid emanations at Milos where As(III) and As(V) constitute ~ 49 and 9% of total As concentration, respectively, with various As sulfides making up for the remainder (Ruiz-Chancho et al. 2013). Taken together, As sulfide formation controls dissolved As content in sulfidic, low pH settings (Cullen and Reimer 1989; Giovannelli et al. 2013; Smedley and Kinniburgh 2002; Henke et al. 2009; Dekov et al. 2013; Price et al. 2013a, b; Ruiz-Chancho et al. 2013; Gilhooly et al. 2014; Godelitsas et al. 2015). In the presence of sulfide, dissolved thioarsenic As sulfides species are formed (Wilkin et al. 2003; Stauder et al. 2005; Henke et al. 2009) and have been shown to constitute ~ 40% of the hydrothermal emissions on the coast of Milos Island (Ruiz-Chancho et al. 2013). This observation provides an important explanation for the higher total As concentration recorded in the white-capped sulfide-rich sediments (Fig. 3e).

The orange-yellow sediments that are typically located in the center of the sampled transect, were absent at time of sampling. These have been described to be composed mainly of As sulfides (Gilhooly et al. 2014) on the basis of their easily recognizable bright orange and yellow colors. However, recent chemical characterization of these sediments, reveal elemental and impure sulfur (Godelitsas et al. 2015). The white patches are famously associated with white elemental sulfur produced via bacterial oxidation of sulfide (Gilhooly et al. 2014). Our results show that the white-capped sediments are sulfide-rich and precipitate typical sulfide minerals. Moreover, the brown-capped sediments appear to also contain a significant amount of sulfide that decreases with depth. Further, sediment physicochemical conditions and thermodynamic constraints suggest conditions appropriate for the formation of the typical orpiment-like minerals common in the sulfide-rich settings on the HVA (Kilias et al. 2013; Giovannelli et al. 2013; Price et al. 2013a; Ruiz-Chancho et al. 2013; Gilhooly et al. 2014). For example, empirical and theoretical studies link major orpiment production in shallow hydrothermal sediments to local temperatures of < 100 °C and reducing conditions, along a wide *Eh*–*pH* range (Breuer and Pichler 2013; Dekov et al. 2013). The formation of realgar (As_4_S_4_) occurs occasionally at T = 25 °C, within a narrower *Eh*–*pH* range (Dixit and Hering 2003). Porewater temperatures in the white-capped patches at Spathi Bay average 62-80 °C relative to a maximum of 81 °C for the orange and yellow patches (Price et al. 2013b). Moreover, the white deposits have a porewater H_2_S content in the range of 0.31–2.89 mM, pH 4.8–5.7, compared to H_2_S levels of < 0.01–1.96 mM and pH 5.2–5.8 in the orpiment-rich orange and yellow sediments (Giovannelli et al. 2013). The precipitation of pyritic minerals in the white-capped sediment (Fig. 4a) is expected to scale linearly with As sulfide, arsenopyrite and arsenian pyrite formation (Cullen and Reimer 1989; Smedley and Kinniburgh 2002; Henke et al. 2009). Also, linear dissolution of orpiment along an increasing pH gradient coupled to oxidation by Fe(III)(oxyhydr)oxides (Lengke et al. 2009), likely accounts for the restriction of orpiment closer to the acidic-sulfide-rich-Fe(III)(oxyhydr)oxide-poor vents. For example, pH increases from ~ 5 close to the vents to pH 8 in the peripheral non-hydrothermally active sediments rich in Fe(III)(oxyhydr)oxides (further discussed below).

By coupling As speciation data obtained by X-ray Adsorption Near Edge Spectroscopy (XANES) to theoretical models, it was proposed that sulfide to Fe(III)(oxyhydr)oxide ratios in sediments and aquifers control As mobility (O’Day et al. 2004). The high sulfide system apparently drove the precipitation of orpiment (O’Day et al. 2004). Further, orpiment production coincides with high reactive S/Fe ratio settings because elevated H_2_S concentrations stabilize orpiment (O’Day et al. 2004; O’Day 2006). In these habitats, As sulfides form more rapidly relative to Fe(III)(oxyhydr)oxides (O’Day et al. 2004; O’Day 2006) and above a certain reactive S/Fe ratio threshold, orpiment becomes saturated in the aqueous phase (O’Day et al. 2004; O’Day 2006). The latter view provides additional justification for the specific correlations seen between porewater As and S in the high sulfide sediment. It also justifies the strong porewater As to Fe linear correlation in the sulfide-poor-Fe(III)(oxyhydr)oxide-rich sediments relative to the sulfide-rich-Fe(III)(oxyhydr)oxide-poor settings.

### Porewater arsenate, arsenite and phosphate geochemistry

As discussed above, Fe(III)(oxyhydr)oxides are expected to become a strong As and phosphate sink away from the white-capped deposit. In the white-capped deposit, sulfide minerals will predominate As scavenging, but not phosphate burial, because sulfide minerals do not typically bind phosphate (e.g., Poulton and Canfield 2006; Brock and Schulz-Vogt 2011). Moreover, in Spathi Bay, we observed an increasing linear correlation between porewater As and Fe concentrations moving outwards from sulfide-rich white-capped sediments. In the sand-capped reference sediment we observed a maximum covariation between As and Fe of 93%. This suggests that Fe(III) (oxyhydr)oxides are only likely to become a potent As sink when sulfide concentrations are low. Consistent with this observation, dissolved phosphate concentrations in Spathi Bay were highest in the sulfide-rich sediments and lowest in the Fe(III)(oxyhydr)oxide-rich deposits.

**Fig. 5:**
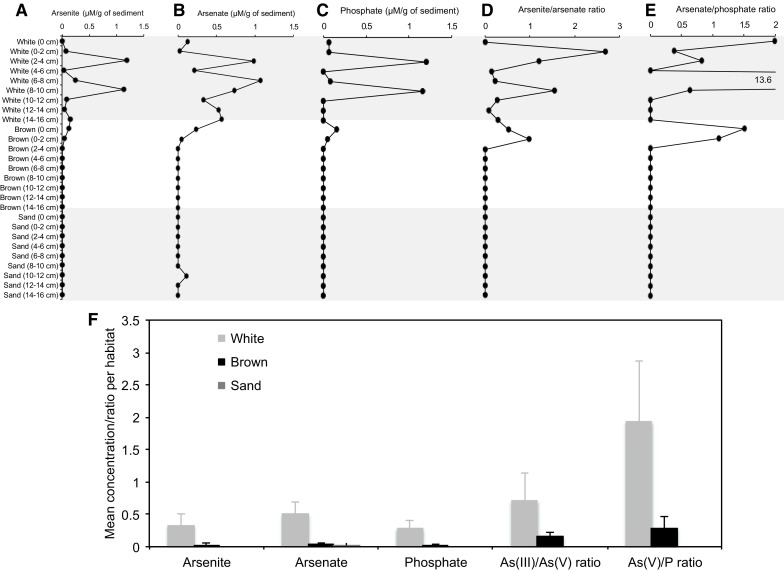
Arsenate, arsenite and phosphate distribution in sediment porewater. **a** Arsenite. **b** Arsenate. **c** Phosphate. **d** Arsenite/Arsenate ratio. **e** Arsenate/phosphate ratio. **f** Mean concentration, arsenite/arsenate and arsenate/phosphate ratios. *Bars* standard deviation from the mean. Concentrations are per gram of sediment

Porewater As(III), As(V) and phosphate content agree with total As dynamics quantified by ICP-OES; i.e., being variable and significantly elevated in the white-capped sediment porewater compared to the brown-/sand-capped deposits (Fig. 5a–c; Table S2). Prominent but variable As(III)/As(V) ratios suggest the white-capped sediments are the most reducing of the three habitats (Fig. 5d–f), an observation consistent with their location in the hydrothermal venting center and high sulfide content (Fig. 3d). Comparably, porewater As(V)/phosphate ratios are generally higher in the white-capped sediment, but negligible across to the sand-capped deposit (Fig. 5e–f).

Phosphate is essential for all life and is believed to have regulated primary productivity through Earth’s history (Reinhard et al. 2017). Crucially, most shallow submarine hydrothermal vent fluids are generally depleted in P, implying that unlike As, hydrothermal fluids are not a major P source but rather a strong seawater P sink (Wheat et al. 1996; Edmonds and German 2004; Poulton and Canfield 2006; Hawkes et al. 2014). Indeed, significant variation in As and P concentrations through Earth’s geological history have been linked to changing levels of submarine hydrothermal influence, Fe(III)(oxyhydr)oxide precipitation and sulfide content (Poulton and Canfield 2011; Chi Fru et al. 2015a; Reinhard et al. 2017; Chi Fru et al. 2016a; Hemmingsson et al. 2018). Importantly, shallow submarine hydrothermal activity in the modern oceans are suggested to account for up to 57% of global hydrothermal P removal from the ocean via Fe(III)(oxyhydr)oxide precipitation (Hawkes et al. 2014). This is a significant value, given that shallow submarine hydrothermalism represents < 10% of global hydrothermal fluid emission on the modern seafloor (Hawkes et al. 2014).

Our results provide important support for quantitative removal of P by Fe(III)(oxyhydr)oxides in shallow submarine hydrothermal systems, hinting for the first time that the As-impacted fluids create severe porewater P deficiency in the affected sediments. Despite the total sediment As concentrations being over 40 times above P, most P is preferentially bound to Fe(III)(oxyhydr)oxides. This observation is in agreement with previous studies that experimentally predicted that As enhances the affinity of P for Fe(III)(oxyhydr)oxides (Chi Fru et al. 2016b; Hemmingsson et al. 2018). Importantly, the lack of variability of this reactive Fe associated P across habitats (Table 1), indicates dissolved P is undersaturated at all sites analysed. P being below the detection limit of 2.0 ppb in the overlying seawater P source, is in support of this hypothesis, as seawater P and dissolved sediment content are tightly linked. Further, the correlation of total sediment P with total sediment As concentrations (Fig. 3e) is consistent with the view that dissolved As impacts P scavenging by Fe(III)(oxyhydr)oxides (Chi Fru et al. 2016b). Unlike Fe(III)(oxyhydr)oxides, sulfide minerals are not known to bind phosphate (Poulton and Canfield 2006; Reinhard et al. 2017). This behaviour is consistent with the superior levels of phosphate detected in the Fe sulfide-rich white-capped sediment porewaters (Fig. 5c), relative to the brown-/sand-capped sediments rich in Fe(III)(oxyhydr)oxides. Data therefore suggest sulfide promotes dissolved P bioavailability by stimulating preferential precipitation of Fe sulfides instead of the Fe(III)(oxyhydr)oxide phosphate sink.

In agreement with Hawkes et al. (2014), we confirm that hydrothermal Fe(III)(oxyhydr)oxides are key scavengers of P in As-rich shallow submarine hydrothermal sediments, demonstrated by efficient binding of P when dissolved As concentrations are experimentally increased (Chi Fru et al. 2016b). It is possible that precipitated P is recycled into porewater through the reduction of Fe(III)(oxyhydr)oxide by sulfide and the Fe-reducing bacteria. For instance, Fe-reducing bacteria in the lineage *Geobacteraceace* (Röling et al. 2014) are prevalent in the sediments together with sulfide.

### As and P cycling gene distribution

The highly conserved function and expression of the *aoxB*, *arrA*, *arsB*, *acr3*-*1* and *acr3*-*2* genes in phylogenetically and metabolically unrelated prokaryotic lineages, is determined primarily by environmental As content. The *pst* gene is required for survival in geothermal environments experiencing high As(V) to phosphate ratios (e.g., Elias et al. 2012). We record similar high As(V) to phosphate ratios in the white-capped sediments, which decline dramatically in sediments tested across the sampling transect through the brown-capped deposit to the sand reference (Fig. 5e). Collectively, this evidence indicates that the abundance of the *aoxB*, *arrA*, *arsB*, *acr3*-*1*, *acr3*-*2* and *pstB* genes in nature is largely dependent on environmental As concentration and less on phylogenetic affiliation.

**Fig. 6:**
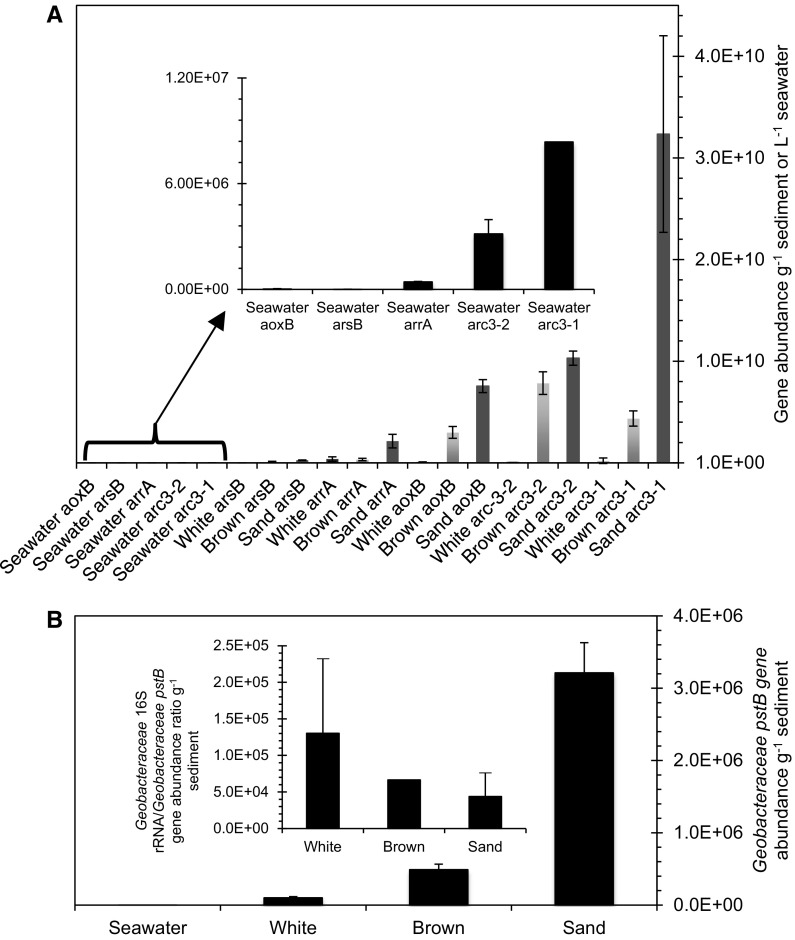
Average arsenic and high affinity phosphate gene abundance in sediment and seawater. **a** Average gene abundance for the periplasmic *aoxB*, *arsB* and *acr3*-*1* and *acr3*-*2* and *arrA* genes. **b** *pstB* gene abundance specific for the *Geobacteraceae*

Thus, the well-constrained *Geobacteraceae arrA*, *acr3*-*1*, *acr3*-*2* and *pstB* gene models are used to track the distribution of these genes across the sampled transect on the basis that shifts in the content of the *Geobacteraceae*-specific genes should correlate with changes in sedimentary As dynamics and whole microbial community composition and abundance. The *aoxB*, *arrA, acr3*-*1, acr3*-*2, pstB* and *Geobacteraceae* 16S rRNA gene concentration, increased from the hydrothermal centers to the sand-capped sediments (Fig. 6), coinciding with a similar increase in whole community 16S rRNA gene abundance from the hydrothermal centre to the sand reference (Callac et al. 2017). Moreover, metagenomic data in review elsewhere are consistent with the systematic increase in microbial community composition and functional gene abundance from the white-capped sediment to the peripheral sand-capped habitat. The metagenomic analysis also indicated that genes involved in As(V) respiration and As(III) oxidation are dramatically low, being below detection limit in a majority of samples. Similar to the qPCR data in study, As detoxification genes predominated the metagenomic library, with *acr* gene content being the most prevalent in the sediments.

Importantly, the metabolically versatile *Geobacteraceae* use a variety of mechanisms to reduce Fe(III) and Mn(IV) oxides and can also oxidize S (Röling 2014). In addition, they are capable of expressing *arrA*, *arsB, acr3*-*1*, *acr3*-*2*, and the *pst* genes, which were all targeted and quantified in this study (Giloteaux et al. 2013; N’Guessan et al. 2010). Fe oxides and S compounds also show strong zonation across the sampled transect (Figs. 3, 4; Table 1). This is consistent with the distribution of sediment *aoxB* gene content, *Geobacteraceae*-specific As cycling *arrA*, *arsB*, *acr3*-*1 and acr3*-*2* genes, together with the *Geobacteraceae*-specific 16S rRNA (Fig. S2) and whole microbial community 16S rRNA genes used for phylogenetic reconstruction (Callac et al. 2017). Mn(IV) oxides are actively being precipitated in the different sediment types (Callac et al. 2017). Thus the *Geobacteraceae arrA*, *arsB, acr3*-*1*, *acr3*-*2*, *pstB* and *Geobacteraceae* 16S rRNA gene distribution, effectively maps over the mineralogical and chemical map of the three distinct sediment habitats. This distribution is relevant for testing our central hypothesis that S and Fe mineralogy are crucial for the distribution of As and P cycling genes in hydrothermal sediments.

**Fig. 7:**
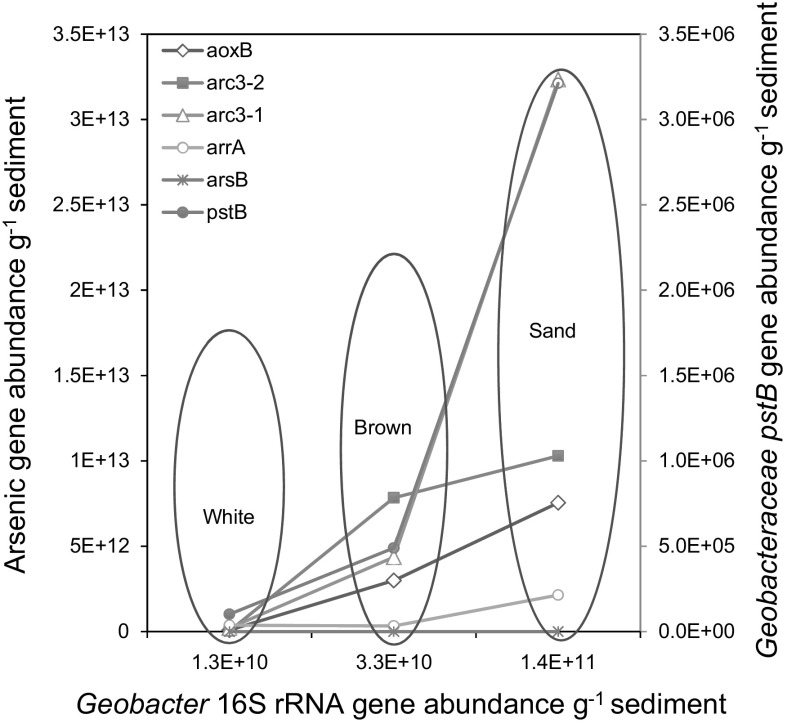
*Geobacteraceae* 16S rRNA gene abundance against As and high affinity phosphate uptake genes. Linear fit with R^2^ values of 0.98403, 0.96795, 0.91712, 0.84513, 0.84194 and 0.73033 for *aoxB*, *arsB*, *acr3*-*2*, *acr3*-*1*, *pstB* and *arrA*, respectively

Most of the genes show a general high content in the first 6 cm sediment depth, with the lowest values encountered in seawater (Fig. 6a). Average sediment As cycling gene content decreased in the order *acr3*-*1 *> *acr3*-*2 *> *aoxB *> *arsB *> *arrA*. According to habitat, As cycling gene density decreased from the sand-capped sediment, through the sediment covered by the brown deposit, to the centrally located white-capped sediment. Seawater had the lowest concentration of As-cycling gene density. A similar trend was observed for the distribution of the *Geobacteraceae*-specific *pstB* genes (Fig. 6b). A plot of *Geobacteraceae* 16S rRNA gene distribution against the various As-cycling and *Geobacteraceae*-specific *pstB* genes shows a correlation among all seven genes (Fig. 7). However, the ratio of the *Geobacteraceae*-specific 16S rRNA gene abundance to the *Geobacteractereace*-specific *pstB* density (inset Fig. 6b) is inversely related to *Geobacteraceae pstB* gene content for each habitat (Fig. 6b). Finally, the *Geobacteraceae*-specific *pstB* and As-cycling gene content exhibit an inverse, correlation with sedimentary sulfide content (Fig. S3), implying a positive relationship with the Fe(III)(oxyhydr)oxides that increased in the opposite direction to sulfide (Fig. 3d, f). The several orders of magnitude lower content for all seven genes in the seawater column, relative to sediment, suggests in situ biogeochemical processes are responsible for generating the observed sediment variability. The data also imply that the metabolic processes controlled by these genes play a more important role in sediment biogeochemistry than seawater.

Temperatures in the studied systems are known to consistently decrease up the sediment profile and away from the hydrothermal centers. For example, sediment temperatures of ~ 80 to 100 °C at ~ 12 cm depths in the vent centers typically drop rapidly upwards, reaching ~ 24 to 35 °C at the seafloor (Brinkhoff et al. 1999; Sievert et al. 1999, 2000). At a distance of 4 m from the emission centers, temperatures fall to ~ 21 °C at ~ 12 cm, with little or no upward variability (Brinkhoff et al. 1999; Sievert et al. 1999, 2000). As already mentioned above, pH increases from < 5.0 to ~ 8.0, away from the hydrothermal loci. Therefore, it is possible that temperatures and pH might account for the differences seen in As and *pstB* gene abundance, going from the white-capped sediments in the hydrothermal center through to the peripheral habitats, located ~ 4 m away. For example, microbial cell counts tend to decrease vertically upwards and increase laterally away from the centers of emission on the seafloor (Sievert et al. 1999, 2000; Giovannelli et al. 2013). However, the ratio of whole community *Geobacteraceae*-specific 16S rRNA gene content to *Geobacteraceace pstB*-specific gene abundance is inversely related to the *Geobacteraceae pstB* gene density per habitat (Fig. 6b). This important observation suggests that *Geobacteraceae* 16S rRNA gene abundance is decoupled from *Geobacteraceae*-specific community *pstB* gene composition. That is, the *pstB* gene content does not scale positively with the *Geobacteraceae* cell density present in each setting. This would be expected if genetic abundance simply reflects increasing cell population density controlled by favourable habitat conditions away from the extreme hydrothermal centers.

The latter assumption is based on the fact that 16S rRNA genes are inherited vertically and because increasing favorable environmental conditions correlate positively with environmental 16S rRNA gene content (Lee et al. 2009). Consequently, specific 16S rRNA gene abundance for targeted phylogenetic groups of microorganisms in an environmental sample, scale linearly with cell count after taking into account the copy number of 16S rRNA genes (Lee et al. 2009). The *Geobacteraceae* family-specific *pstB* gene data was therefore normalized to *Geobacteraceae* family-specific 16S rRNA gene abundance, since the latter is expected to scale with local total *Geobacteraceae* cell density. On the other hand, the *Geobacteraceae*-specific *pstB* gene is expected to scale with the subset of *Geobacteraceae* community members containing high affinity phosphate uptake specific genes in their genome. The data suggest that the increasing sediment *Geobacteraceae*-specific *pstB* gene abundance, known to respond to P limitation (N’Guessan et al. 2010), was not related to a general increase in *Geobacteraceae* population density away from the hydrothermal loci. Therefore, the data imply that increasing habitable conditions related to temperature and pH, with distance away from the hydrothermal vents, are not the main factors underlining the enrichment of *pstB* genes along the studied transect.

As expected, the paired variables of *Geobacteraceae*-specific 16S rRNA gene content and the individual genes, validate the fact that the *Geobacteraceae*-specific 16S rRNA genes, together with the measured genes, follow the established trend of total sediment gene content increasing from the hydrothermal  center to the reference sand (Callac et al. 2017). The data should therefore not be interpreted to mean that the *Geobacteraceae* are the sole contributors of the genes analysed in the sediments. For example, the R^2^ value between *Geobacteraceae*-specific 16S rRNA gene content and the *pstB* gene is 0.84194. This R^2^ value equates to a ~ 84% positive variation between the two variables, suggesting that ~ 16% of the detected *Geobacteraceae*-related lineages along the sampled transect might not necessarily carry the *pstB* gene. This correlation could be attributed to the strong variability of phosphate abundance in the different habitats. Similarly, the > 90% positive variation between the *Geobacteraceae*-specific 16S rRNA gene content and the arsenic detoxification genes, *aoxB*, *arsB* and *acr3*-*2*, suggests that the sediments enrich a *Geobacteraceae* assemblage with strong resistance against As toxicity in all three habitats. Consistent with this view, it has been observed that the *Geobacteraceae* respond to increasing As content via the up-regulation of As detoxification genes in the *acr3* gene family (N’Guessan et al. 2010). Importantly, the *acr3* gene content, which is the most abundant As detoxification gene in the sediments, covaries positively with the *Geobacteraceae* family 16S rRNA gene abundance (Fig. 7).

### Control on As and P cycling gene enrichment

The low As resistance gene content in the seawater column is several orders of magnitude lower than sediment concentrations (Fig. 6a). This suggests that As stress in seawater is negligible relative to the sediments. It also indicates that the overlying seawater microbial communities may be more vulnerable to sudden As perturbation than those inhabiting the sediments. Moreover, As precipitation by Mn oxides, Fe(III)(oxyhydr)oxides and particularly As sulfides (Cullen and Reimer 1989; Smedley and Kinniburgh 2002; Maity et al. 2005; Henke et al. 2009; Kilias et al. 2013; Godelitsas et al. 2015), are suggested as effective processes preventing dramatic contamination of seawater by hydrothermal As-rich fluids. This, in addition to dilution of the hydrothermal fluids by seawater, likely accounts for the lower seawater As(V) and As(III) respiratory reductases and oxidases content in comparison to the sediments. The extraordinary predominance of As(III) extrusion genes in the sediments (Fig. 6a), further hint that respiratory As(V) and As(III) energy conservation are likely overwhelmed by As detoxification (resistance). As detoxification is suggested to be most likely intense in the sand sediment, where reactive S/Fe ratio minima coincide with As resistance gene maxima. The low high affinity phosphate uptake gene concentration in seawater, despite the fact that dissolved P content is equally limiting, is likely related to the fact that the *Geobacteraceae* thrive well when oxygen is absent. As demonstrated by our data, seawater was well ventilated.

Several quantitative studies have shown that microbial adaptation to phosphate availability depends on lineage, population size, nutrient requirements and rates of metabolism (e.g., Jansson 1993; Van Mooy et al. 2006, 2009; Karl 2014; Lin et al. 2016). High affinity phosphate uptake predominates when As(V)/Phosphate ratios rise above a certain threshold (Elias et al. 2012) and/or when P levels are extremely low, regardless of whether As(V) is present or absent (Thiel 1988; Takahashi et al. 1990; Jansson 1993; Dyhrman and Haley 2011; Guo et al. 2011; Karl 2014; Lin et al. 2016). The significant decrease in As(V)/phosphate ratio from the white to the sand reference (Fig. 5e, f), suggests that the low P content encountered in the brown-/sand-capped sediments drives the enrichment of high affinity P uptake genes away from the white capped-sediment. If the As(V)/phosphate ratio were important, then one would naturally expect high affinity P-uptake gene abundance to peak in the white-capped sediments that contain As(V)/phosphate ratios that are > 1 and several orders of magnitude above those in the brown and sand-capped sediments. The generally lower microbial population density in the white-capped sediments also implies less competition for nutrients, including P, compared to the brown and sand-capped sediments that are progressively enriched in microbial abundance (Callac et al. 2017). Therefore, the dissolved P content coupled to microbial abundance, drives the enrichment of high affinity P uptake genes in the sediments over the As(V)/phosphate ratio.

**Fig. 8:**
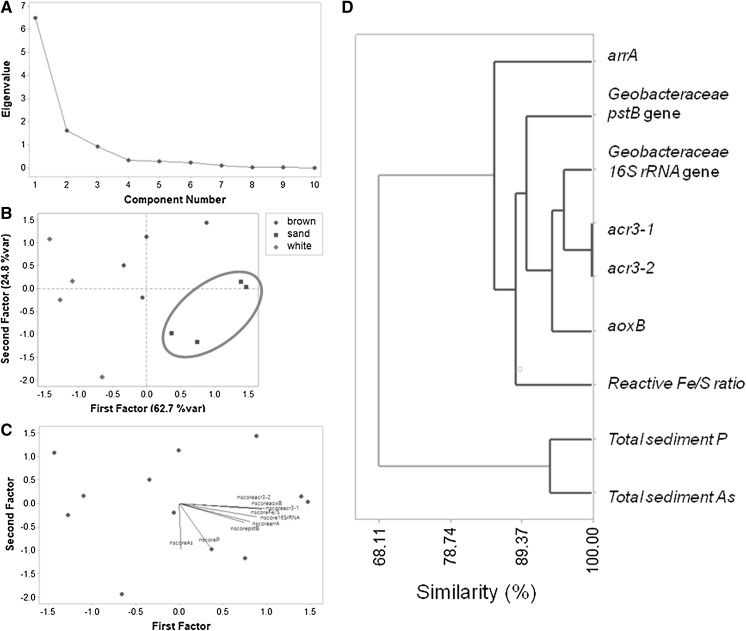
Principal component analysis (PCA). Plots are for selected parameters, As respiration, As resistance and high affinity phosphate uptake genes, total sediment P and As and reactive Fe to S ratios. **a** Screen plot showing a maximum of three-factor analysis possible for the dataset. **b** Three-factor PCA analysis of the correlation matrix plotted against habitat (see Table S3 for calculated variables). **c** Sediment biplot. **d** Complete linkage-correlation distance similarity dendrogram

Results of factor analysis (Table S3) provide evidence that the changing Fe/S ratio is closely related to the genomic presence of the studied As- and P- genes. It is noted that the *arsB* gene was not considered because of its low abundance in all types of sediment (Fig. 6a). Specifically, a two-factor solution accounting for 87.5% of the total variance is identified based on the scree plot criterion (Fig. 8a–c). The changing reactive Fe/S ratio is grouped with the *Geobacteraceae pstB* gene, the predominant As(III) efflux pump *acr3*-*1* and *acr3*-*2* genes, the respiratory As(V) reductases (arrA) and As(III) oxidases (aoxB) in Factor 1 (62.7% of total variance). Total sediment P and As are grouped independently in Factor 2 (24.8% of total variance) (Table S3). There is a clear separation of the sand-capped habitat from the white-/brown-capped sediments (Fig. 8b). Ιt should be emphasized that the robustness of factor analysis is limited by the dimensionality of the data determined by the availability of a sufficient number of samples for the number of variables (Reimann et al. 2008). However, the grouping of variables is further supported by the cluster analysis results which are presented graphically in a dendrogram (Fig. 8d). The similarity axis represents the degree of association between the variables, the greater the value the more significant the association. There is a corresponding high similarity (> 85.35%) between reactive Fe/S ratios and the studied genes, while Total sediment P and As form a separate cluster of high similarity level (> 93.75%) (Fig. 8d). Studies tracking the expression levels of the specific *Geobacteraceae pstB* genes by rtqPCR relative to P bioavailability, suggest the PstB proteins are switched on under similar P-limiting conditions, as reported in this study (N’Guessan et al. 2010). Moreover, in situ respiratory *arrA* genes were concomitantly unregulated, together with the *acr3* As resistance genes, in the *Geobacteraceae,* following increase in dissolved As content (N’Guessan et al. 2010; Giloteaux et al. 2013).

## Conclusions

**Fig. 9:**
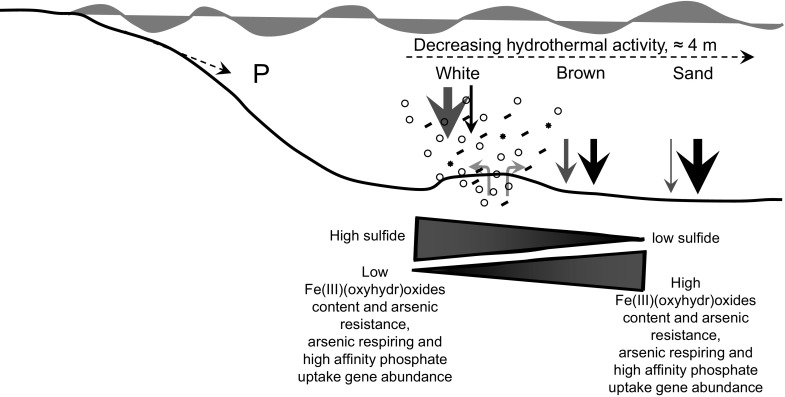
Conceptual model for As and P cycling along the sampled transect. The size of the red and black arrows are proportional to the quantity of As sulfides and Fe(III)(oxyhydr)oxides precipitated and stored in sediments. Open circles represent hydrothermal gas bubbles
- The sediments and seawater contain disproportionately lower As-respiring gene content relative to As resistance genes, suggesting that As detoxification is crucial for survival over As metabolism.
- The predominance of As detoxification genes, especially *acr3* genes encoding the environmentally widespread Acr3 As(III) extrusion protein, implies the sediment community harbors a tremendous capacity for rapid response to sudden As perturbation.
- The distribution of high affinity phosphate uptake genes is suggested to be dependent on sediment pore phosphate concentration, which in turn is controlled by Fe(III)(oxyhydr)oxides content and scavenging.

This study, focused on the coast of Milos Island, Greece, presents a comprehensive biogeochemical model for the fate of As and P in As-rich shallow submarine hydrothermal ecosystems, on the coast of Milos Island, Greece, demonstrating that:

- Quantitative regulation of dissolved porewater As and P concentration by sulfide and Fe(III)(oxyhydr)oxide minerals during sediment–seawater interaction, produce nutrient-deficient porewaters containing < 2.0 ppb phosphorus.
- Reactive S to reactive Fe ratios may be critical in determining the distribution of genes required for As resistance, As respiratory and high affinity phosphate uptake in As-rich shallow submarine hydrothermal sediments (Fig. 9).

## Electronic supplementary material

Below is the link to the electronic supplementary material.

Supplementary material 1 (DOCX 1055 kb)
