# Supplementary material for: Arsenic and high affinity phosphate uptake gene distribution in shallow submarine hydrothermal sediments
Source: Biogeochemistry. 2018 Sep 20;141(1):41–62. doi: 10.1007/s10533-018-0500-8 (PMC6413627; doi:10.1007/s10533-018-0500-8)
Supplement: Supplementary file 1 — Supplementary material 1 (DOCX 1055 kb) [file 10533_2018_500_MOESM1_ESM.docx]

Supplementary dataset

**Arsenic cycling and high affinity phosphate uptake gene distribution in shallow submarine hydrothermal sediments**

Ernest Chi Fru^1,2*^, Nolwenn Callac^1,2^, Nicole R. Posth^3^, Ariadne Argyaki^4^, Yu-Chen Ling^2^, Magnus Ivarsson^5^, Curt Broman^1^, Stephanos P. Kilias^4^.

^1^Stockholm University, Department of Geological Sciences and Bolin Centre for Climate Research, SE-106 91, Stockholm, Sweden.

^2^Cardiff University, School of Earth and Ocean Sciences, Park Place, CF10 3AT, Cardiff, Wales, UK.

^3^Nordic Centre for Earth Evolution (NordCEE), Department of Biology, Campusvej 55, 5230 Odense M, Denmark.

^4^National and Kapodistrian University of Athens, Department of Geology and Geoenvironment, Panepistimiopolis Zographou 157 84 Athens, Greece.

^5^Department of Palaeobiology and Nordic Centre for Earth Evolution, Swedish Museum of Natural History, Stockholm, Sweden.

| **Gene** | **Strain used for curve calibration** | **Primer name** | **Primer sequence** | **Primer concentration (nM)** | **Annealing temperature°C** | **Reference** |
| --- | --- | --- | --- | --- | --- | --- |
| ***arrA*** | *Geobacter sulfurreducens (DSMZ 12127)* | qArr1F | 5’- GATCCACGCTTCTCCACCTC-3’ | 500 | 60 | Giloteaux et al. 2013 |
|  |  | qARR1R | 5’-CCCCGGCTTTAAAGAGGTTC-3’ | 500 |  |  |
| ***aoxB*** | *Marinobacter Santoriniesis(DSM 21262)* | *aoxBM1-2F* | 5’-CCACTTCTGCATCGTGGGNTGYGGNTA-3’ | 500 | 60 | Quéméneur et al. 2010 |
|  |  | aoxBM2-1R | 5’-GGAGTTGTAGGCGGGCCKRTTRTGDAT-3’) | 500 |  |  |
| ***arc3-1*** | *Geobacter sulfurreducens (DSMZ 12127)* | qA1-3F | 5’-ATGGCCAGCTCGAAaTTGTT -3’ | 500 | 60 | Giloteaux et al. 2013 |
|  |  | qA1-2R | 5'-GCGATGGCCAGCTCRAARTTRTT-3' | 500 |  | Fahy et al. 2015 |
| ***arc3-2*** | *Rhodoferax ferrireducens (DSMZ 15236)* | qA2-1F | 5’-GGCCAGTTCGAAGAAGTTGG-3’ | 500 | 60 | Giloteaux et al. 2013 |
|  |  | qA2-1R | 5’-GCCGATTTTGATCCAGGTGT-3 | 500 |  |  |
| ***arsB*** | *Desulfovibrio tunisiensis (DSMZ 19275)* | darsB1F | 5'-GAACATCGTCTGGAAYGCNAC-3' | 500 | 53 | Poirel et al., 2013 |
|  |  | darsB1R | 5'-GTACACCACCAGRTACATNCC-3' | 500 |  |  |
| ***pstB*** | *Geobacter sulfurreducens (DSMZ 12127)* | pstB-310F | 5′-CCGTTCCCCAAATCGATCT-3′ | 500 | 60 | N’Guessan et al. 2010 |
|  |  | pstB-422R | 5′-ATGGCGGCGTTAGTGAGG-3′ | 500 |  |  |
| **16S rRNA geobacteraceae** | *Geobacter sulfurreducens (DSMZ 12127)* | GEO-494F | 5′-AGGAAGCACCGGCTAACTCC-3’ | 500 | 60 | Homes et al. 2002 |
|  |  | GEO-825R | 5‘-TACCCGCRACACCTAGT-3‘ | 500 |  | Andersson et al. 1998 |

Table S1. Genes, pure culture standards and primers used for qPCR analysis. With the exception of the *Geobacteraceae* *pstB* and 16S rRNA-specific gene primers, the remainder of the primers are universal (see the references for the choice of primers).

Table S2. Sediment porewater and seawater chemical analysis in ppm.

Table S3. Total variance explained and matrix of Varimax rotated factor loadings and communalities for normal score transformed geochemical and genomic data. Significant positive and negative factor loadings are indicated in red numbers.

Variable Factor1 Factor2 Communality

nscoreAs 0.020 -0.974 0.949

nscoreP 0.345 -0.919 0.964

nscoreFe/S 0.870 -0.090 0.765

nscoreaoxB 0.936 -0.119 0.891

nscorepstB 0.805 -0.391 0.800

nscoreacr3-2 0.980 -0.106 0.972

nscoreacr3-1 0.980 -0.106 0.972

nscorearrA 0.738 -0.402 0.707

nscore16SrRNA 0.883 -0.282 0.858

Variance 5.6455 2.2313 7.8768

% Var 0.627 0.248 0.875

Fig. S1. Distribution of Fe, P and As in sequential Fe extractions. W, B and S, represent white, brown and sand-capped sediments, arranged according to sediment depth. (a) Fe quantified in different Fe(III)(oxyhydr)oxide phases. (b) As content associated with different Fe(III)(oxyhydr)oxide phases. (c) P content associated with different Fe(III)(oxyhydr)oxide phases. (d) Total sediment Fe, As and P to total Fe(III)(oxyhydr)oxide ratios.

Figure S2. Abundance of the As cycling genes *aoxB*, *arsB*, *arrA*, *arc3-2* and *arc3-1* and high affinity phosphate uptake *pstB* genes with sediment depth (cm) and seawater water depth (m). (A) Sand-capped sediment. (B) Brown-capped sediment. (C) White-capped sediment. (D) Seawater. Data not represented were below detection limit.


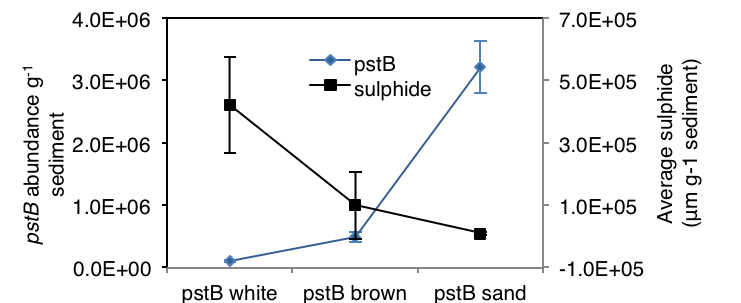

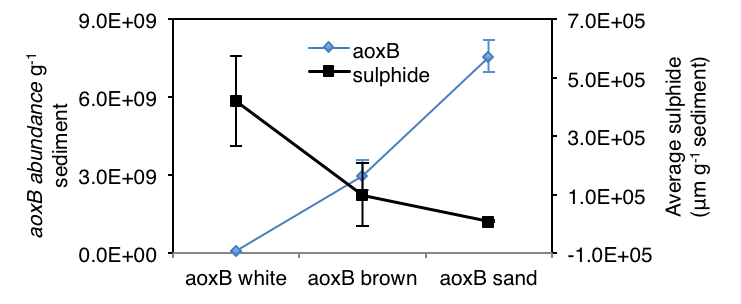

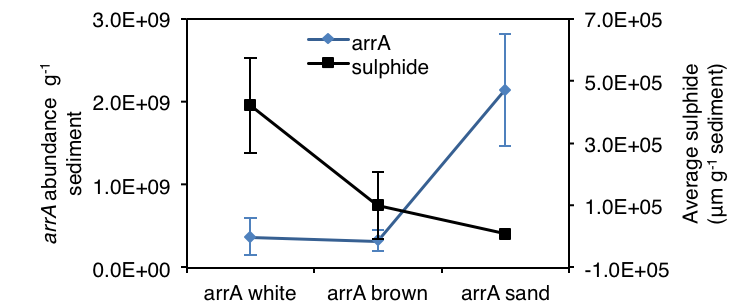

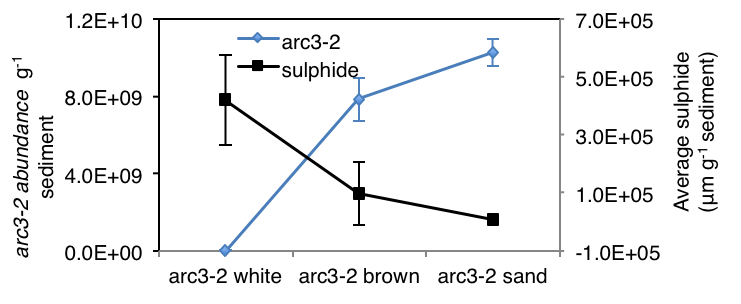

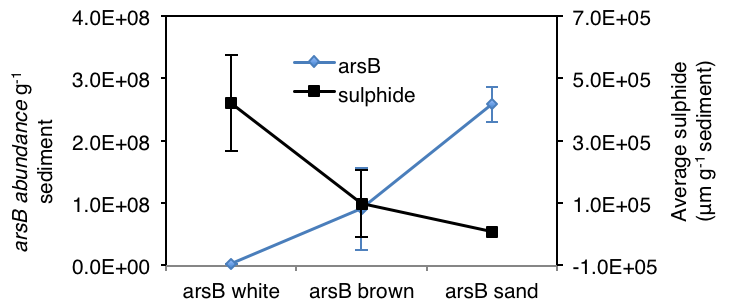

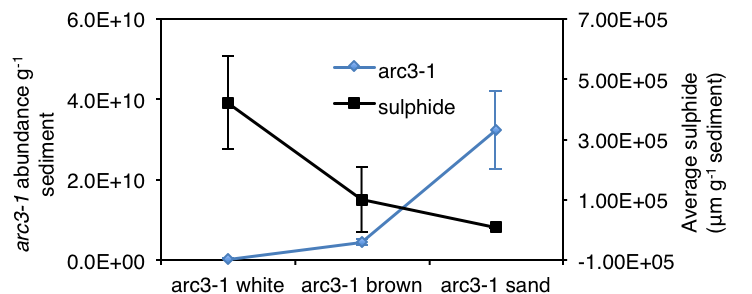


(A)

(B)

(C)

(D)

(E)

(F)

Fig. S3. Relationship between sulphide and distribution of As and high affinity phosphate genes in the first 20 cm of sediments at Spathi Bay, Milos Island. Values are average core values for the entire sediment depth for two replicate cores per habitat type. Scale bars for the sulphides are standard deviations across a 20 cm depth profile, while bars on the gene graphs are errors for measurements averaged across the same depth profiles.

**References**

Anderson RT, Rooney-Varga JN, Gaw CV, Lovley DR (1998) Anaerobic Benzene Oxidation in the Fe(III) Reduction Zone of Petroleum-Contaminated Aquifers. Environ Sci Technol 32:1222-1229.

Fahy A et al*.* (2015) 16S rRNA and As-Related Functional Diversity: Contrasting Fingerprints in Arsenic-Rich Sediments from an Acid Mine Drainage. Microbial Ecol 70:154-167.

Giloteaux L et al. (2013) Characterization and transcription of arsenic respiration and resistance genes during in situ uranium bioremediation. The ISME J 7:370-383.

Holmes DE, Finneran KT, O'Neil RA, Lovley DR (2002) Enrichment of Members of the Family Geobacteraceae Associated with Stimulation of Dissimilatory Metal Reduction in Uranium-Contaminated Aquifer Sediments. Appl. Environ. Microbiol. 68:2300-2306.

N'guessan AL et al (2010) Molecular analysis of phosphate limitation in Geobacteraceae during the bioremediation of a uranium-contaminated aquifer. The ISME J 4:253-266.

Poirel J, Joulian C, Leyval C, Billard P (2013) Arsenite-induced changes in abundance and expression of arsenite transporter and arsenite oxidase genes of a soil microbial community. Res Microbiol 164:457-465.

Quéméneur M*, et al.* (2010) Population structure and abundance of arsenite-oxidizing bacteria along an arsenic pollution gradient in waters of the Upper Isle River Basin, France. *Appl. Environ. Microbiol.* **76**, 4566-4570 (2010).
